# Community perceptions about factors influencing access to care after sexual violence in North Kivu, Democratic Republic of the Congo: a qualitative study

**DOI:** 10.1186/s13031-025-00662-4

**Published:** 2025-04-05

**Authors:** Hanna Reinholdz, Jack Palmieri, Helena Frielingsdorf, Esther Katungu Kalere, Gérard Nteziryayo Heritier, Meggy Verputten, Anette Agardh

**Affiliations:** 1 Department of Clinical Sciences, Division of Social Medicine and Global Health, Lund University, Malmö, Sweden; 2 Médecins Sans Frontières, Operations Centre Amsterdam, Amsterdam, Netherlands; 3 Department of Health, Medicine and Caring Sciences, Division of Society and Health, Linköping University, Linköping, Sweden; 4 Médecins Sans Frontières, Operations Centre Amsterdam, Goma, Democratic Republic of the Congo

**Keywords:** Gender-based violence, Sexual abuse, Sexual assault, Rape, Help-seeking behaviour, Democratic Republic of the Congo

## Abstract

**Background:**

Sexual violence is widespread in the eastern parts of the Democratic Republic of the Congo, including in the North Kivu province. Moreover, in this region survivors of sexual violence often have limited access to care and encounter a variety of barriers when seeking care and support. The aim of this study was to explore community perceptions about access to care, barriers, enablers and possible actions to improve access to care for survivors of sexual violence in North Kivu. A deeper understanding of community perceptions about access to care can guide ongoing efforts to overcome barriers and increase access to care for survivors of sexual violence.

**Methods:**

The study utilised a qualitative design, based on focus group discussions with male and female adult community members in the study area. Previous experience of sexual violence was not a requirement. The transcripts from the discussions were analysed using manifest and latent qualitative content analysis.

**Results:**

A total of 18 focus group discussions were carried out. The analysis resulted in three main themes; Knowledge and misconceptions around medical consequences crucial for care seeking, Community and family attitudes playing a dual role in care seeking behaviours and Care seeking dependent on optimised healthcare facilities and sensitive staff.

**Conclusions:**

Lack of correct knowledge, harmful attitudes from community and healthcare staff, and poorly adapted healthcare services constitute barriers to accessing care. Improved awareness raising around sexual violence is needed to address both lack of knowledge and misconceptions. Efforts should be made to build upon the community support models and actively work to improve community attitudes towards survivors of sexual violence. In addition, there is a need for better adapted healthcare services with improved proximity, access for different groups of survivors and respectful and well-trained healthcare staff.

## Introduction

The Democratic Republic of the Congo (DRC) has been plagued by several conflicts and wars that have destabilised the country and heavily impacted both individuals and communities. North Kivu province, located in the eastern DRC, is a particularly conflict-torn area. Both sexual violence (SV) and violence perpetrated by intimate partners are widespread in DRC [1, 2]. Especially high rates of sexual violence in the wake of the conflicts has been observed in the eastern parts of DRC, where according to a survey-based study, 40% of women and 24% of men have reported experience of sexual violence [3]. Even though conflict-related sexual violence in DRC is widely acknowledged [4–6], it is important to recognise that the sexual violence in DRC can not solely by attributed to militarised conflict. A qualitative interview study by Alexandre et al. showed that sexual violence was common in eastern DRC before the start of the war in 1996, and that sexual violence was embedded both in the gender norms placing women in a subordinate position and in perceptions around masculinity [7].

Sexual violence is defined by WHO as “any sexual act, attempt to obtain a sexual act, unwanted sexual comments or advances, or acts to traffic, or otherwise directed, against a person’s sexuality using coercion, by any person regardless of their relationship to the victim, in any setting, including but not limited to home and work” [8]. Survivors of sexual violence are at risk for both medical and psychological sequelae [9]. Examples of immediate medical consequences from sexual violence are sexually transmitted infections (STI), including HIV, and physical injuries such as anal tears. Medical consequences specific to female survivors include unwanted pregnancies, unsafe abortions and vaginal tears. Psychological consequences for both male and female survivors include, but are not limited to, drug and alcohol abuse, anxiety, depression and post-traumatic stress disorders [10, 11]. There is also an increased risk of death through suicide and as a result of HIV infection [12]. In addition to the medical and psychological consequences, both female and male survivors risk negative social consequences such as stigmatisation, shame and rejection by family, partner and/or community which may further exacerbate negative outcomes [11, 13, 14].

Timely access to medical and psychosocial care for survivors of sexual violence is important. Some parts of the care package offered to survivors are time sensitive which makes timely access to care crucial. HIV post-exposure prophylaxis is only effective if provided within 72 h of the violence, and emergency contraception is only effective up to 120 h after the violence [15]. Both of these prophylactic measures are more effective the earlier they are provided. However, both medical care and psychological support can be provided after these time limits, such as post-exposure prophylaxis or treatment for other STIs, safe abortion care, testing and treatment for HIV, care for injuries, and psychological support [16]. In addition to medical and psychological care, survivors also need to be offered social support, including economic support, protection and legal aid, based on their individual needs and preferences and the options available in the context [17].

Despite the importance of timely care, a number of barriers exist that challenge care seeking behaviours. A study performed between 2004 and 2011 using Demographic and Health Survey data in 24 low-income countries showed that only 40% of women who had experienced sexual violence disclosed the event to someone, and only 3% sought medical care [18]. Among women who had experienced intimate partner violence (IPV), the numbers were even lower and only 2% of them had sought care [18]. Some of the barriers reported were embarrassment, the belief that there is no use in reporting, and normalisation of violence [18].

In DRC, one study from 2008 based on household surveys and facility assessments from the Kasongo health zone showed that 58.6% of survivors of sexual violence sought medical care and that 46.1% of those who sought care did so within 72 h [19]. Barriers to accessing care for survivors have been reported from studies conducted in the DRC using a variety of methods, i.e. household surveys and individual interviews [20], focus group discussions [21] and register based data [22]. The barriers found include shame, stigma, lack of sensitive care providers, lack of knowledge of services available, lack of quality in the services available, fear that family would find out, rejection by family/husband, lack of means to access care and that survivors would wait for physical symptoms to develop before seeking care [20–22]. Only one of these studies included participants from North Kivu [20]. Barriers for male survivors of sexual violence to access care in South Kivu have also been reported in a qualitative study and include fear of social exclusion, fear of being seen as homosexual, lack of services adapted for male survivors, lack of trust in healthcare providers’ confidentiality, and cost of transportation [23].

Even though the prevalence and consequences of sexual violence in DRC have been relatively well studied, there is little research that focuses on the region of North Kivu. Thus, there is still a knowledge gap regarding specific barriers to healthcare access and how community members perceive survivors’ need for healthcare, barriers to care and how access to care can be improved. Even though some barriers may be common to many contexts, local variations exist, and it is crucial that each programme offering care and/or support to survivors of sexual violence learns from the communities themselves and understands the contextual barriers and enablers in order to optimise access to care.

In our previous file-based study of care seeking patterns and factors influencing timely care seeking among survivors of sexual violence in North Kivu, we found that age, sex and referral pathways impacted timely care seeking and that the majority of survivors accessing care did so in the clinics specialised in sexual violence care, sexual and reproductive health and mental health support [24]. Male survivors were more likely than female survivors to seek care within 72 h, and all age groups younger than 50 years old were more likely to seek care within 72 h compared to those above 50 years. Further, survivors who were referred by the community, a family member, a mobile clinic or authorities were less likely to seek care within 72 h, compared to those who sought care on their own, without a referral [24]. To further explore the reasons behind these findings and to understand how access to care can be improved, the aim of the present study was to explore community perceptions about access to care, barriers, enablers and possible solutions to improve access to care for survivors of sexual violence in North Kivu.

## Methods

A qualitative study design was used, using focus group discussions (FGDs) conducted with community members in the study area. The primary research question was the following: what are community members’ perceptions regarding barriers and enablers for care seeking, timely access to care and how access to care can be improved for survivors of sexual violence?

### Study setting and population

Data collection was conducted in the catchment area of two Médecins Sans Frontières (MSF) programmes located in Walikale and Mweso, North Kivu, DRC, between 14th June and 6th September 2019. In both study areas, MSF in collaboration with the Ministry of Health offers care to survivors of non-partner sexual violence and sexual, physical, and emotional intimate partner violence. This care is offered in hospitals, health centres and through three ‘Tumaini’ clinics. The Tumaini clinics (‘hope’ in Swahili) are healthcare facilities that offer care to survivors of SV and IPV integrated with care for STIs, contraceptive counselling and provision, and psychosocial support. Within the two project areas there have also been awareness-raising activities about SV and IPV, both in healthcare facilities and communities. These have been carried out by MSF health promotion and community engagement teams working together with local Ministry of Health community health workers so-called “Relais Communautaires”.

### Sampling and data collection

Within the study area, 11 villages/towns of different sizes were selected through convenience sampling, based on being places where the research team was able to go within the study period, due to logistic and security challenges. In each location, purposive sampling was used to sample participants who could provide information on the topic of interest. As we aimed to explore general perceptions around access to care for survivors of sexual violence, participants were not required to have previous experience of sexual violence and there were no questions around this in the recruitment process. Men and women over the age of 18 who were healthy enough to participate in a focus group discussion were included. Focus groups were organized separately by gender, with men and women allocated to separate focus groups. Efforts were made to keep the age range similar within the focus groups, although this was not always possible. Those under the age of 18, not healthy enough to participate in a focus group discussion or not able to give informed consent were excluded. Participants in each village/town were selected through gatekeepers (e.g. male and female community/village leaders from each location), who informed potential participants about the study purpose. Those who were willing to participate were then provided with more information by the research team before being asked to provide consent. The FGDs were performed in the village/town from which the participants were recruited, in a relatively private area such as the home of a village leader, under a tree or under a roof built as a “community area”. With the help of community leaders, we ensured that non-participants stayed away from that area, to create a space as private as possible. We aimed to obtain rich and high-quality data (information power) and did not aim for a complete description of all aspects of the studied phenomenon [25].

The FGD topic guide was based on the preliminary results from our previous quantitative study of file-based data concerning survivors’ access to care within the two MSF programmes [24] and was developed in collaboration with the Congolese research team. The topic guide was pre-tested with a group of MSF staff, including both men and women from the study area. The guide was developed in French and translated into Swahili with backtranslation into French. No written translation of the guide into Kinyarwanda was deemed necessary, as persons who are literate are literate in Swahili. When Kinyarwanda was needed, the facilitators interpreted during the session. The topic guide included questions and prompts addressing perceptions around sexual violence and consequences, help and health seeking behaviours, barriers and enablers to access care and solutions to increase access to care for survivors. It also included questions about community-based care, methods for health promotion messaging and perceptions around medical certificates for sexual violence, albeit these topics were not included in the present study and will be address in a separate manuscript. The topic guide was specifically designed to not encourage sharing of own traumatic experience, but rather to focus on community response and reactions and used vignettes to provide a context within which the topic was discussed. A story of a woman experiencing sexual violence by an unknown perpetrator was told to the participants and discussion was centred around that story. Then the participants were also encouraged to discuss what would have happened if the perpetrator would have been the husband of the survivor, if the survivor would have been a man and if the survivor would have been a child. No definition of sexual violence was provided in the focus group discussions, to allow for the definitions and perceptions of the participants to emerge during the course of the discussions. A flexible participatory technique was used, where all the main topics were covered in all discussions, but the facilitators allowed a natural flow of the discussion. Flexible prompts were used when necessary to encourage the discussions [26].

The FGDs were led by authors HR, EKK or NHG (EKK and NHG being Congolese nationals) and were conducted in Swahili or Kinyarwanda. The research team, including the FGD leaders, note takers and translators, included both males and females and two females were non-Congolese. All members of the research team received training in qualitative research, interview techniques, psychological first aid and the study protocol by HR. The FGD leaders all had extensive experience of working with survivors of sexual violence and sexual violence programming within the study context, and also had prior experience of conducting focus group discussions on the topic. Live interpretation between Swahili or Kinyarwanda and French was done by a member of the research team, to allow for understanding by non-Swahili/Kinyarwanda speaking research team members. All discussions were audio-recorded and later transcribed and translated to French by a trained transcriber/translator.

## Data analysis

The data was analysed using manifest and latent qualitative content analysis as described by Graneheim and Lundman [27]. Transcripts were read through in French several times by HR to get a sense of the content before being translated into English by HR. Meaning units linked to the aim and research questions of the study were identified in 3 transcripts and condensed by HR. These were then verified by AA, and when consensus was reached, HR identified and condensed meaning units for the remaining transcripts. HR then conducted the coding with continuous discussions with AA. When consensus was reached for the coding, HR developed the sub-categories, categories and themes, with continuous feedback from AA and JP. Consensus regarding the final analytical model, where the sub-categories and categories represent manifest content and the themes represent latent content, was achieved through discussions with all authors. The qualitative data analysis software NVivo version 14 was used for the analysis [28].

### Ethical considerations

This study followed the ethical principles outlined in the Helsinki declaration [29]. Since the topic of interest is sensitive, the study team also paid particular attention to ethical issues associated with research on sexual violence, in order to ensure the physical, psychological and social well-being of the participants throughout the study. This was done in accordance with the WHO recommendations on research in the field of sexual violence [12].

Prior to asking for consent to participate in the study, the study was explained in detail, including the objectives, risks, benefits and voluntary nature of participation. Written information was provided in Swahili and oral information offered in Kinyarwanda and Swahili. Persons who are literate can read in Swahili as Swahili is the language used in schools and therefore it was deemed sufficient to translate the written information to Swahili. Individuals were asked to explain in their own words the implications of participating, giving the facilitators a chance to assess if they had fully understood the information provided. If an individual was illiterate, the facilitators ensured that the information sheet was read and explained to the individual and they were offered to ask a third party of their choosing to read for them. Participants were specifically informed about the limitations to confidentiality in focus group discussions, were asked to not share information outside the group discussions and informed that no results where an individual could be identified would be presented. They were also informed that they could withdraw their participation up to the point of publication. Written consent was obtained from all participants and no compensation was provided for participation.

The research team received training in psychological first aid and how to handle sharing of sensitive information or traumatic events by the participants. Participants were offered to speak with members from the research team in private after the group discussion if they would like. A referral pathway was put in place through MSF programmes, for both medical care and psychological support if needed by any participant in the study. Psychosocial support was also ensured for the research team, through the MSF staff health system.

All data has been stored according to the data protection guides of MSF. Audio-files and transcripts do not include any personal identifiable data and have been stored with limited access only to the primary investigator (HR) and the study coordinator (MV). All data will be destroyed 5 years after the finalization of the study.

## Results

A total of 18 FGDs were conducted, in 11 different locations, 9 in locations within the Mweso programme area and 9 in locations within the Walikale programme area. The number of participants in each FGD ranged from 5 to 10 and a total of 138 individuals participated, 81 women and 57 men. Ten of the FGDs were with female participants and 8 with male participants, and all participants were over 18 years. In most locations there was one FGD conducted with men and one with women; however, in two villages it was only possible to conduct FGDs with women. The median length of the discussions was 62 min. One discussion was ended prematurely due to insecurity in the area which forced the research team to leave early.

**Fig. 1:**
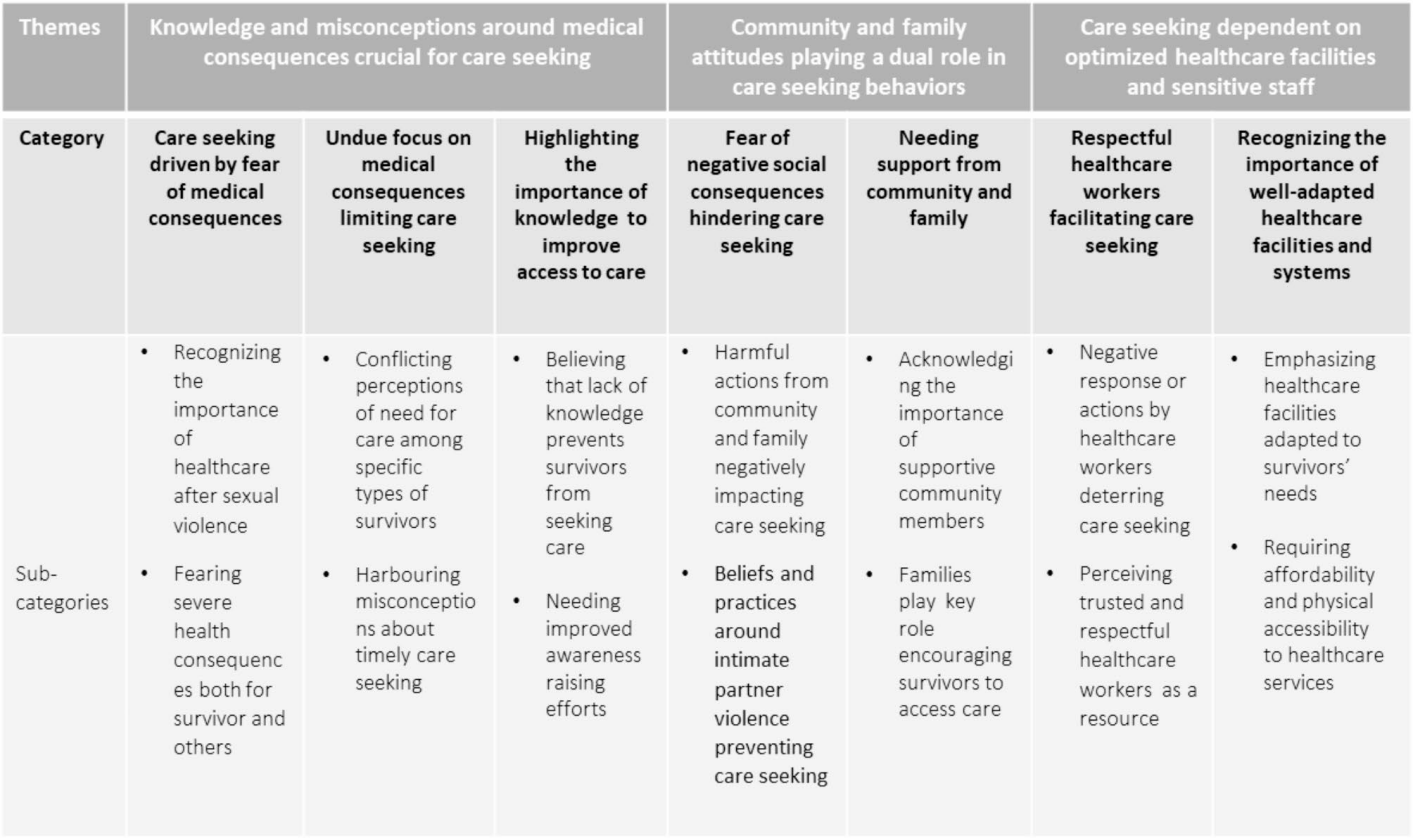
Analytical model presenting the themes, categories and sub-categories

The analysis resulted in three themes: Knowledge and misconceptions around medical consequences crucial for care seeking, Community and family attitudes playing a dual role in care seeking behaviours and Care seeking dependent on optimised healthcare facilities and sensitive staff. These themes are based on seven categories and 14 sub-categories. The overall analytical model is presented in Fig. 1.

The following section presents each theme together with its supporting categories and sub-categories. Quotes from the FGDs are used to further illustrate the sub-categories. Participants are identified by the number of the FGD and whether the participants in the group were female or male.

### Knowledge and misconceptions around medical consequences crucial for care seeking

The first theme describes how knowledge, and the lack thereof, impacts care seeking behaviours in different ways. Seeking care after an experience of sexual violence was perceived as an important action and fear of medical consequences was identified as motivating care seeking. However, the medicalised view of sexual violence, together with misconceptions around intimate partner violence, male survivors, and timing of care seeking, could also lead to survivors not seeking care and support. Lack of knowledge about consequences of sexual violence was identified by the participants as one of the main barriers for survivors to seek care, and to address this, the need for improved awareness-raising activities in the community was highlighted.

#### Care seeking driven by fear of medical consequences

Both female and male participants recognised to a great extent the need for healthcare after an incident of sexual violence and this was linked to an expressed fear of the negative medical consequences of sexual violence, mainly HIV and other STIs.

#### Recognising the importance of healthcare after sexual violence

When asked what a person would do after experiencing sexual violence, participants mentioned seeking healthcare as the most important action, both for men and women. Often it was presented as the one thing the survivor should or had to do, rather than what they actually would do. This indicates that the participants were aware of the need for medical care, but that they also recognised that many survivors would not seek care.

Participants mentioned that seeking care was important in order for survivors to be tested for infections, receive preventive care for infections and pregnancies or receive treatment for diseases. The need to prevent diseases was mentioned in both male and female focus group discussions whereas the need to prevent pregnancies was only mentioned in female focus groups. The need for testing and prevention of STIs was specifically highlighted throughout the discussions, even though other consequences were mentioned.

*She might be contaminated by sexually transmitted diseases that she doesn’t even know of. She has to go to the clinic to receive care and to know if she was contaminated or not*,* and if she is not contaminated she can prevent the consequences.* (FGD 12, male)

Even though a great deal of focus in the discussions concerned the importance of medical care, the need for psychological support or counselling was also acknowledged by both men and women.

*If Rose [an imaginary survivor of sexual violence] keeps it a secret*,* it might happen that she will feel tormented and even get a depression or psychological signs*,* but if she goes to a healthcare structure she might receive care and psychological support.* (FGD 11, female)

There was also a sense of belief in the healthcare being effective, both in terms of prevention or treatment of diseases and with regard to a more general sense of promoting wellbeing.

*[…] there are many here who are already victims of sexual violence*,* many are already victims of many diseases following sexual violence and the care really helps us a lot; otherwise we would all be sick.* (FGD 11, female)

#### Fearing severe health consequences both for survivor and others

There was a strong sense of fear among the participants, both males and females, especially about transmission of HIV and other STIs. Moreover, some participants meant that if a survivor does not get treatment they might die, illustrating this sense of fear.

*There are those who are raped and who tell themselves “as no one saw me*,* I can keep quiet about this”*,* even though she might have caught a sexually transmitted infection which will then lead to her death.* (FGD 13, female)

The fear of adverse medical consequences was also identified by participants themselves to be one of the main motivators for survivors to seek care.

*The fear of the possible diseases you can get during sexual violence can make the person go and seek care quickly.* (FGD 17, female)

In addition to the consequences for the survivor, participants also highlighted the fear of spreading STIs to partners and how that could motivate care seeking. This was mentioned in both female and male groups. It was also mentioned that a survivor should think about the potential consequences for her children if she does not seek care.

*[…]she needs to think about her two children*,* so that they don’t become orphans; she should seek care for the sake of her children.* (FGD 13, female)

#### Undue focus on medical consequences limiting care seeking

While knowledge of medical consequences could drive care seeking, the strong focus on the physical consequences of SV, especially on infections, in combination with misconceptions about male survivors, survivors of IPV and the time window for care seeking, could also prevent survivors from accessing care. Having some knowledge but not understanding the full extent of medical and psychological consequences or the care package that can be offered to survivors could thus constitute a barrier for care seeking.

#### Conflicting perceptions of care needs for specific types of survivors

There were conflicting perceptions about the need for care for female survivors of sexual violence perpetrated by an intimate partner. Some participants felt strongly that survivors of sexual IPV do not need any care as there would be no need to prevent infections.

*If it was my husband who raped me*,* I can’t go to a healthcare structure because it is my husband. I trust him*,* and he can’t transmit any diseases to me.* (FGD 13, female)

Others believed that female survivors of sexual IPV could seek care, but only if they were actually experiencing physical symptoms. Yet others argued that female survivors of sexual IPV should always seek care, either because they would need psychological support, because their partners could have an infection or because of the risk of an unwanted pregnancy.

*You can be raped by your husband*,* and if you are not feeling well*,* then you can always seek care. But if you are not feeling unwell*,* you can’t seek care.* (FGD 17, female)

The perceptions about care needs also varied with regard to male survivors. Even though many participants, both males and females, thought it would be important for males to seek care as they could also have medical consequences, some female participants argued that male survivors would not have the same consequences as female survivors, or no consequences at all, and therefore would not need healthcare.

Some participants looked at it from the survivor perspective and said that there were men who would not acknowledge their need for care and therefore would not seek care.

*The man can be raped and say to himself “for me*,* there are no consequences*,* because I’m not a woman” and yet there are diseases. He can also say “me*,* I’m not a woman who can get an unwanted pregnancy” and imagine that there are no diseases*,* and that might make him stay at home.* (FGD 15, female)

#### Harbouring misconceptions about timely care seeking

Participants were generally aware of the need for timely care, and it was repeatedly mentioned by both female and male participants that survivors should seek care immediately or as soon as possible. In some discussions the window of three days or 72 h was mentioned, but there were also participants who suggested that survivors have to seek care within one day, two days or five days.

*She has to go to the clinic just after the aggression. If she is raped in the fields or in the plantation*,* she should not even go back home; she has to go directly to the clinic for care*,* as soon as possible.* (FGD 12, male)

Participants, both male and female, insisted that survivors *had* to seek care within a specific timeframe and that there would be no use seeking care if they could not make it within that limit. This was strongly related to the perception of prevention of infections being the only care or the only important care which could be provided, and that if a certain time had passed it would be too late for preventive care and thus no benefit to seeking care at all. The perception of the need for timely care seeking and the focus on STI prevention could thus become a barrier to access care for those who for different reasons could not seek care within the timeframe. However, even though some participants felt strongly that seeking care after a longer period of time was unnecessary, others recognised the importance of seeking care at any given time, highlighting that there would be both medical and psychological healthcare needs that could still be addressed.

*- From my point of view*,* she can’t [seek care after 5 months]*,* she is already infected and it is possible that she has already infected her husband. Now*,* after 5 months*,* what can she look for at the healthcare structure? It doesn’t make sense. She might already have infected her husband.*

*- I think that she can go after 5 months*,* even after a year. As she has been raped*,* she is afraid and she is unstable. She can talk to the nurse and the nurse can orient to a service like the mental health services*,* and if it is necessary*,* they can provide her with medications.* (FGD 15, 2 females)

#### Highlighting the importance of knowledge to improve access to care

Even though participants themselves had an extensive understanding of the need for medical care, they thought that there were many in their surroundings who did not have this knowledge and perceived this as a major barrier to access care. When discussing how access to care could be improved, both female and male participants brought up the need for more awareness-raising activities as an important intervention.

#### Believing that lack of knowledge prevents survivors from seeking care

Both male and female participants expressed that one of the main reasons for both male and female survivors to refrain from seeking care was lack of information about sexual violence and the consequences, as well as where and how to seek care.

*If she keeps this as a secret and doesn’t inform anyone*,* that can lead to her not accessing the care. And all that is because she has not been informed or educated with regards to sexual violence or on how to behave in case of sexual violence.* (FGD 12, male)

Similarly, being well informed about sexual violence and the medical consequences was perceived as enhancing care seeking for both male and female survivors.

*Now*,* with the sensitisation from the nurses and the health promoters*,* we know that after rape*,* we should go to the health centre. Before there were no messages like this and we would only stay at home.* (FGD 4, female)

With regard to the awareness-raising messages there was also a strong focus on the physical consequences, and a belief that if a survivor knows about the possible consequences, and most importantly the risk of infections, such knowledge will drive them to seek care.

#### Needing improved awareness-raising efforts

Continued and increased awareness-raising activities were highlighted by both male and female participants as one of the most important interventions to increase access to care. Enhancing knowledge and awareness among the population was suggested to reinforce the current health promotion activities. Increasing the number of people conveying messages about sexual violence in the community was considered as crucial to ensure that the information would have a broader reach. There was a request for both specific health promoters and to use community health workers (Relais Communautaires) for this purpose. The necessity of outreach to far-away areas was also pointed out.

*Personally*,* if I was raped I can go to the Tumaini clinic*,* but there are several who have never heard the sensitisation sessions around sexual violence. You have to add or multiply the health promoters in several areas and in the far-away areas in order for everyone to be sensitised.* (FGD 11, female)

When asked how access to care for male survivors could be improved, it was mentioned in both female and male groups, that awareness raising with messages specifically targeting men, explaining that consequences can happen to them as well, were needed.

*For example*,* during the sensitisation*,* when you meet a man you can share with him about the sexual violence*,* explaining to him that the consequences can happen to everyone. If you share with this man he can think about seeking care if he is victim sexual violence.* (FGD 14, male)

An additional aspect of awareness raising targeting men concerned the need to address men’s attitudes towards their wives who have been raped, which could then increase women’s access to care. This was brought up in both female and male groups, both as a response to questions about who a survivor could talk to after experiencing rape, and as response to questions about how access to care can be improved for female survivors.

*Even the men have to be sensitised*,* to tell them not to kick their wives out because they have been victims of sexual violence.* (FGD 13, female)

### Community and family attitudes playing a dual role in care seeking behaviours

Negative reactions and harmful actions from community and family members were perceived by both male and female participants as strong barriers to disclosure and care seeking. In addition, participants also mentioned practices and beliefs that could prevent survivors from seeking care. On the other hand, community and family members were also perceived as facilitating access to care. Overall, this shows the importance of the attitudes, norms, beliefs and practices within the community for care seeking behaviours among survivors.

#### Fear of negative social consequences hindering care seeking

Participants described many harmful and stigmatising actions by community and family members, specifically by husbands, towards female survivors of SV. It was evident that participants, both men and women, perceived that the fear of these negative consequences was a main reason for female survivors to abstain from disclosing the event and/or from seeking care.

#### Harmful actions from community and family negatively impacting care seeking

Harmful and stigmatising actions directed towards female survivors within the community described by the participants included being called a prostitute, being discredited in the community and being mocked.

*Here*,* if a woman is victim of sexual violence*,* when she comes to the community*,* everyone starts to make fun of her and booing her.* (FGD 9, male)

For male survivors the negative social consequences mentioned were the risk of conflict, either between families or within the family and in one male and one female focus group it was mentioned that men or boys being raped would be seen as a sorcery.

Not being able to get married was mentioned by male participants as a negative social consequence and for young women and girls, it was mentioned in both female and male focus that they would lose value after being raped as they would no longer have the status as “virgins”.

*Those around her might stigmatise her*,* and in addition to that*,* if she wasn’t married*,* she can’t get married. So*,* the information can’t be shared in the community*,* it has to stay a secret for her. She needs to go to the clinic without telling anyone. It can’t be spread in the community.* (FGD 12, male)

In one discussion it was also mentioned that stigmatisation could affect the possibility for a female survivor to earn her living.

*There are also other consequences*,* like if she used to be a shopkeeper*,* now she won’t know how to run her business because wherever she goes*,* people will point at her and say “she’s the woman who was raped”.* (FGD 9, male)

Being a survivor of SV was also perceived as strongly linked to shame which in addition to stigma was described as preventing female survivors from disclosing and seeking care.

*It’s always the shame that is the obstacle for victims to access care. Because she knows that if she talks about it*,* her story will be shared with the whole village.* (FGD 4, female)

Shame was also mentioned as a barrier to disclose or to seek care for male survivors. This was mentioned by both male and female participants. In one female group discussion, it was however mentioned that males would not feel ashamed and could thus easily seek care.

*The biggest consequence for a man is the shame*,* the shame of being raped by another man like him. Instead of sharing with someone he will decide to seek care directly*,* without sharing with anyone else. He will just explain to the nurse who will keep it confidential.* (FGD 5, male)

In addition to the more general stigmatisation by community members, for female survivors the fear of negative reactions by the husband was particularly highlighted. These reactions included not believing the survivor and insulting or physically hurting the survivor. One of the strongest fears seemed, however, to be the fear of being kicked out or rejected by the husband, causing a divorce or in other ways destroying the household. This was mentioned in both female and male focus groups.

*Another barrier*,* there are women who live with their husbands who are very mean*,* and that can lead to the women not seeking care*,* that she doesn’t dare to talk about the aggression*,* related to the fear of her husband. And as the husband is mean*,* the women can stay in the house by fear of divorcing the husband. She will judge it better to stay at home with her disease rather than having the household destroyed.* (FGD 14, male)

The fear of being exposed as a survivor in the community was also apparent in the strategies described by the participants as being employed by both male and female survivors when seeking care. Different strategies about how to seek care without people around them knowing were discussed in both male and female focus groups, including how to behave in the healthcare facility and how to make up an excuse to the husband or family to be able to visit the health centre without them knowing.

*[…] it is not easy to say that you are a victim of sexual violence. For example*,* when you arrive at the Tumaini you say that you are sick*,* that you have an STI or that you have a secret disease. Because it is really hard for someone to say that they have been victim of sexual violence. In the Tumaini it is a secret with the nurse*,* so there you can say that you are a victim of sexual violence*,* but outside of the consultation room*,* there you can only say that you are victim of an STI. The secret stays between me and the nurse.* (FGD 11, female)

#### Beliefs and practices around intimate partner violence preventing care seeking

Perspectives were raised with regards to beliefs or practices within the community which could prevent care seeking and this was particularly mentioned in relation to sexual violence by an intimate partner. One example is the belief that a matter like IPV should stay within the family and not be shared with anyone outside.

*Yes*,* it exists [intimate partner violence]*,* but there is nothing to do when it happens in our home*,* as if you announce it*,* the man will beat you or the neighbours will tell you that you are sharing a family secret.* (FGD 3, female)

Another belief that was brought up was that sexual violence by an intimate partner to some extent should be accepted by the woman as sexual relations are part of a marriage and that a rape by a husband cannot be compared to a rape by another aggressor. This was expressed both by female and male participants and linked to the belief that a female survivor of sexual IPV should not seek care.

*I think that she can’t seek care [if raped by her husband] because it is something that she is used to going through in the marriage. Because the sexual intercourse that she has had with the husband*,* it is not like an intercourse with an aggressor*,* it is like the intercourse she is used to in the marriage*,* that is why I think that she can’t seek care.* (FGD 14, male)

In addition, private “solutions” between the partners were put forward by both male and female participants as a more appropriate action than seeking healthcare. Another type of solution mentioned was that the survivor should talk to the family of the husband or to the elderly in the community so that they could talk to the husband and ask him to stop the violence.

#### Needing support from community and family

Although harmful attitudes and actions from community and family were acknowledged as major issues, the role of the community and family in supporting survivors and encouraging care seeking was also evident.

#### Acknowledging the importance of supportive community members

Lack of support from peers was mentioned as preventing care seeking for female survivors at the same time as the positive role of community members in supporting female and child survivors was emphasised.

*There is a girl in our village who was raped by FDLR. When she came back home she shared with everyone and we oriented her to the healthcare structure. When she came back no one pointed fingers or stigmatised her; instead we felt bad about what had happened to her.* (FGD 5, male)

In several FGDs it was mentioned that female survivors could or would disclose to a community member, and disclosing to a trusted community member was sometimes mentioned as the first thing a survivor would do. It was commonly mentioned that female and child survivors would be oriented or accompanied to the healthcare centre by the person to whom they disclosed, in contrast to the findings mentioned above with regard to stigmatisation. Community leaders, pastors, friends and women in the community were mentioned as potential first points of contact for survivors, and it was mentioned that supporting peers is the responsibility of everyone, not only professionals.

The importance of the support from community members being respectful and discrete was also mentioned.

*And another thing that we can add regarding a woman who is sensitised to go to a health centre*,* I might have oriented her to the healthcare structure but I need to have a sense of confidentiality. I have to keep what she shared with me a secret*,* and she will feel supported; she won’t feel discouraged; she will feel that she is a person like all the others.* (FGD 13, female)

Community health workers were specifically mentioned as trusted members of the community to whom female survivors could disclose and who would also orient survivors to the healthcare structure.

#### Families play key role encouraging survivors to access care

Support from family members was also expressed in both female and male focus groups as important for survivors. Having support from family and particularly from parents was perceived by participants as crucial for child survivors’ access to care. Some mentioned that child survivors would disclose to the parents, and the responsibility of parents to bring the child to care was highlighted. This was particularly mentioned with regards to girls.

*You are a parent*,* it happens that your child*,* 10 or 12 years old*,* this child can tell the parents. If you are the dad or the mom*,* as a parent you will reassure her and tell her to keep it secret and then you can orient her to the healthcare structure.* (FGD 11, female)

However, it was also mentioned by both men and women that child survivors would not talk about their experience, but that parents would see signs or symptoms and understand what has happened and subsequently be able to help the child.

It was emphasised by both male and female participants that it is difficult for children who experience sexual violence to access care alone and that they would have to be brought for healthcare by an adult, most commonly a parent. The parents’ role in informing and educating their children, and particularly girls, around sexual violence was also mentioned.

*It is a bit complicated because for the child it is difficult to decide on her own to seek care or to go to the health centre*,* but an adult can decide herself. The child has to find a person who can orient her to the healthcare and otherwise she might not be able to receive the care.* (FGD 16, male)

For adult female survivors it was mentioned that they could speak to a family member, and especially mothers were mentioned. The husband’s role to support their wives in case they experience sexual violence was also brought up, despite the negative and harmful reactions from husbands mentioned above. In both female and male groups, it was mentioned that a female survivor could tell the husband. A man who would support his wife and help her receive healthcare after being raped was described as a “good man”.

*If the woman is well informed*,* she will tell her husband that what happened was against her will*,* that it was an unknown*,* and if the husband is a good man he will bring her to the healthcare structure for care.* (FGD 7, male)

### Care seeking dependent on optimised healthcare facilities and sensitive staff

The third theme highlights the importance of well adapted healthcare systems and healthcare staff trained for the task. The attitudes and behaviours of healthcare workers were acknowledged as highly influential for care seeking. How the healthcare facilities are set up was also recognised as impacting access to care, as well as the need for affordable and proximal services.

#### Respectful healthcare workers facilitating care seeking

According to both female and male participants, healthcare workers play a key role in influencing care seeking behaviours. Harmful actions by healthcare workers or lack of trust in the healthcare work force were perceived as hampering care seeking whereas ensuring trusted healthcare workers was identified as a way of improving access to care.

#### Negative response or actions by healthcare workers deterring care seeking

Attitudes, behaviours and personal links with healthcare workers were all perceived by participants as factors preventing care seeking among survivors. The fear of not being met with respect or that healthcare workers would not respect confidentiality was expressed.

In addition, the fear of not being believed by the healthcare worker was mentioned in one male focus group as a potential barrier, especially with regard to sexual violence by an intimate partner.

*She can’t seek care because she will not be able to respond to the question of who has raped her when the perpetrator is her husband. The nurse might not believe her if she says that she was raped by her husband.* (FGD 14, male)

#### Perceiving trusted and respectful healthcare workers as a resource

Generally there seemed to be a high level of trust in healthcare workers and the care available both among males and females. Disclosing to a healthcare worker rather than to someone in the community or family was a commonly mentioned help seeking pattern for both men and women.

*She can talk about the problem with a nurse because if she talks to her husband*,* she will first of all have problems with her husband. It is better to talk to a nurse because she will keep the secret and take care of her with confidentiality.* (FGD 14, male)

That healthcare workers could keep confidentiality was perceived as especially important, and ensuring confidentiality was one of the actions suggested for improved access to care. Both male and female participants wanted healthcare workers who were respectful and welcoming, and some female participants preferred to have female care providers.

*It is also good to advise the nurses*,* because if you say that you were raped*,* even before they start to provide care*,* they will start to say that “you were raped because you went to the fields*,* if you had stayed at home you would not have been raped.” It would be better if they were trained to respect someone who has been raped*,* and to care for them without any comments.* (FGD 6, female)

#### Recognising the importance of well-adapted healthcare facilities and systems

The need for a healthcare system that would facilitate care seeking was clear. Ensuring confidentiality and limiting the risk of being exposed as a survivor were important aspects and different models of care responding to that need were discussed. In addition, proximity to service and free healthcare for survivors were perceived as important factors to improve access to care.

#### Emphasising healthcare facilities adapted to survivors’ needs

The importance of confidentiality was not only mentioned in relation to healthcare workers, but also when talking about the healthcare facilities. Having a healthcare facility which is designed to provide both confidentiality and privacy, thereby preventing survivors from being exposed was perceived as important by both males and females. Fear of being exposed when entering a clinic was brought up as a barrier, specifically when talking about the specialised Tumaini clinics.

*There are people who don’t want to seek care*,* especially at the Tumaini clinic*,* as they are afraid that people will see them enter*,* because they are afraid of the interpretations. The population knows that it is there these types of infections are treated and many people have bad thoughts with regard to this subject*,* so they will avoid seeking care at the clinic.* (FGD 12, male)

In one male focus group discussion it was suggested that care of survivors of sexual violence should be integrated with other types of services, for example malaria care, and that having a healthcare facility where only sexual violence care is provided should be avoided. Ensuring that survivors would be treated as a priority when seeking care was also identified as important in both female and male focus groups.

Some female participants expressed that it was easy to access care in the Tumaini structures and that they preferred to receive the care there. The reasons for this included that they had received information about the clinic, that it was close, that the care provision was for free of charge, that survivors would be cared for directly or that the necessary drugs and tests were available there. Some participants also wanted to have more Tumaini clinics implemented, especially those who did not already live close to one.

On the other hand, in one female discussion it was mentioned that the Tumaini clinics had a low acceptance among men and were seen as only open for women. As a solution to address that, it was requested in the same focus group to create separate clinics for male survivors and also to involve men in the development of such clinics.

*Sometimes men are sick. They have STIs. Sometimes you can tell them to seek treatment at the Tumaini*,* and they say that they can’t because it is shameful and that the clinic is for women*,* and as a man they can’t go. Most of them think that the ones who go to the Tumaini clinic are pregnant women and women who have been raped*,* and that is why a man who is victim of sexual violence does not want to go there to receive care.* (FGD 10, female)

#### Requiring affordability and physical accessibility to healthcare services

Long distance to a healthcare facility offering care to survivors was identified as a factor preventing both care seeking in general and timely access to care. This was mentioned in both male and female discussions and with regards to both male and female survivors. Lack of time for care seeking was another potential barrier, brought up in a female focus group.

*The difficulty to access care is also among the barriers. For example*,* you might want to seek care but if you have been raped in the mining areas*,* over there*,* there is no care available and the distance is a barrier as well.* (FGD 6, female)

To overcome the distance barrier, it was recommended by both male and female participants to increase proximity by opening clinics like the Tumainis in more locations or by offering the care in health posts or other structures closer to the survivors. Having community health workers or other focal points in the communities ready to orient survivors to the healthcare facility was also asked for by both male and female participants.

Lack of transport opportunities was also perceived as a barrier and organising transport or paying for transport was proposed as ways to improve access to care.

At the healthcare system level, cost for the care was perceived as a barrier by both men and women. Participants described that in some facilities the care was not available for free and that the lack of free care constituted a barrier for those who could not afford to pay, and also that when the care was indeed free, the population was not always aware of this. Having access to free healthcare, including having the knowledge about it, was perceived as motivating care seeking.

*When the care is free*,* it motivates the patient to seek care*,* but if you don’t have the means to seek care*,* if you have to pay for the care*,* you risk to stay at home and die.* (FGD 6, female)

## Discussion

This study provides new and valuable insight into community members’ perceptions about access to care for survivors of sexual violence in North Kivu, DRC, and how such access can be improved. These results expand and deepen the previous body of knowledge derived from eastern DRC that has mostly focussed on South Kivu. Our findings concerning the impact of negative social consequences and particularly fears of stigmatisation and rejection are in line with previous research from DRC [21, 30–32]. However, that undue focus on physical manifestations may impact care seeking adds new knowledge to the field. The community members’ suggestions that access can be improved through increased awareness, greater proximity to healthcare services, adaptations of healthcare services to create safe access, more training of healthcare workers and solutions for transportation difficulties also add new insights.

No definition of sexual violence was provided to the participants, to allow for their own interpretations and definitions to emerge during the course of the discussions. However, from the discussions it was clear that participants’ perceptions mostly concerned penetrative sexual violence and this should be considered when interpreting the results.

Our findings show that there was a general knowledge about the importance of seeking care that was very much linked to medical consequences, particularly HIV and other STIs. This indicates that awareness raising has been successful in one sense. However, we also found that this focus on medical consequences, combined with some misconceptions, could constitute a barrier to seeking care. More nuanced messaging is needed regarding the benefits of support for survivors, going beyond the medical consequences and prophylaxis for infections. The lack of knowledge about risk of medical consequences for female survivors of sexual IPV and the lack of knowledge about care and support available for them indicates that more dialogue around this is needed. Scott and colleagues have shown in a mixed method study that IPV is highly normalised and accepted in eastern DRC [20]. In contexts where IPV is accepted as normal, it is essential to work actively to ensure that survivors have access to care and support.

Two qualitative studies have shown that access to care for male survivors of sexual violence is particularly limited in eastern DRC [23, 33]. The misconceptions around their need for care as shown by some participants in the current study could be one contributing factor. Stigmatisation of male survivors and shame linked to the gender role of a man and views on masculinity are known barriers to seeking care both globally [34, 35] and in DRC [23, 33]. This was also shown in our findings, even though it was expressed in one female group discussion that men would not feel ashamed as women would.

The focus on medical consequences and the need for timely access to care can also negatively impact access. When the community perception is that one *must* come within a specific time limit after the incident, there is a risk that survivors who cannot access care within this limit will not present for healthcare at all. Even if it is important for survivors to access care within three days in order to be able to prevent HIV transmission, it is also important that survivors who cannot access care within three days still seek and receive care whenever it is possible for them to come. Our previous file-based study showed that care seeking within 72 h was common, especially in the Mweso area [24]. The MSF awareness-raising activities in the area (especially in Mweso) have been highlighting the importance of seeking care within three days, which likely had a positive impact on knowledge about the urgency of seeking care among the population but can also have prevented those who were not able to access care within three days from coming at all. Correct information about which parts of the care package require urgency is important to convey to the target population to enable them to make informed decisions about when to seek care, or if that is not feasible, to use more general messages around seeking care as soon as possible. It is thus important to understand and address misconceptions about timing in any awareness-raising activities and to focus more on the need for both medical care and psychosocial support, which can be provided at any time.

In all groups there was a strong consensus that more knowledge is needed. The need for more knowledge through awareness raising to increase care seeking among survivors of sexual violence has been shown in other studies from humanitarian settings, for example in a survey study among refugees in Uganda [36]. The participants’ recommendation that the messages needs to be spread further and reach those who live far away from the main towns and villages is important to fulfil.

It is also important to design messages that not only focus on the consequences of SV and that might instil fear, but instead to talk about the care and support, including both psychological and social support, that can be provided and how peers and partners can be a support to survivors rather than causing more harm. It is clear from the current findings that awareness-raising activities are appreciated by the communities and should be continued, augmented and adapted to different target groups, including different types of violence.

Many of the social consequences cited in this study are devastating for survivors and constitute a second victimization as well a barrier for seeking help. Stigmatisation and judgement of survivors as well as rejection by family and husband can have severe consequences and further impact the wellbeing of survivors. Stigma and the negative social consequences faced by survivors of SV in DRC have been found in many studies, as well as their association with the decision to avoid seeking care [21, 30–32], which our study further corroborates. That different community members were also mentioned as possible enablers for care seeking shows that there is a strong potential in the use of community as supportive peers rather than stigmatising and further harming survivors. The dual role of the community depicted in the analysis indicates that there is to some extent already a support system within the community and we believe that this can, and should, be built upon. The discrepancy between the attitudes of community members towards survivors, being mostly negative, and the perceptions that community members also can or should support survivors was also noted by Babalola and colleagues [37] who in their study highlight the conflict around descriptive and prescribed norms. Both our results and their findings show the same discrepancy, that community members know that survivors should be treated with respect, but that in reality there are still strong negative perceptions and attitudes towards survivors. However, the recognition within the community that there are negative and harmful attitudes that may result in lack of access to care may also represent an opportunity. This recognition is already a first step towards working actively to further influence attitudes and foster more acceptance for survivors of both SV and IPV and should be further built upon in SV/IPV programming.

Husbands were specifically highlighted as hindering care seeking among female survivors even though they were also mentioned as potential support persons. The importance of a supportive husband needs to be included in work addressing sexual violence in the area. As proposed by participants in this study, there is also a need for specific activities targeting men with the objective of being more supportive of women who are raped. This is crucial to ensure a safe environment where women feel safe to disclose their experiences and access care and support after sexual violence. The importance of key family and community members as well programmes which could help community members, male relatives and husbands to accept survivors of rape has also been discussed in previous studies from South Kivu [21, 38].

Negative and harmful attitudes and how they negatively impact care seeking does not only apply to community and family members but also to healthcare workers. Having trusted health care providers who respect survivors and maintain confidentiality was highlighted as important and shows the need for targeted training to all healthcare providers who are in contact with survivors.

As SV survivors fear negative social consequences, it is important to ensure that they can access care in a safe and discrete way. Rapid and discrete care pathways were highlighted by participants in this study as important measures to improve access to care. Stand-alone services for SV/IPV or specific care provision rooms with only SV/IPV care provision are not recommended in this context for this specific reason. However, integrating services fully within an emergency department or outpatient department would risk comprising privacy and confidentiality and might pose obstacles towards appropriate training and attitudes by the staff, as there will be many competing priorities. Therefore, integration with a few selected services might be a better alternative. However, careful considerations must be taken to ensure all survivors are welcome and different models of care may be needed for different types of survivors, i.e. men, women, and children. The semi-vertical Tumaini model was appreciated by many women but was perceived as being for women only and not welcoming for male survivors. Additional care pathways for male survivors or a more inclusive profile of Tumaini clinics are needed to ensure all survivors of SV have a safe pathway to care. The barriers mentioned on a healthcare structure level also indicate a need for complementary models, closer to the communities as distance was highlighted as a barrier and proximity as an enabler. Offering care for survivors at the health centre and health post level is needed, but likely not sufficient. Offering care within the communities using community-based models, with a trusted community member, possibly a community health worker, can be one way to reduce the distance barrier as well as some of the stigmatisation or risk of exposure [39]. However, community-based care models need to take sociocultural aspects into account and be designed in close collaboration with the community to ensure acceptability and feasibility [40]. Another model to consider is the holistic, one-stop care model, where medical care, psychosocial support, legal aid and socioeconomic support is provided in one location, as the model implemented in the Panzi hospital in South Kivu [41]. Careful consideration needs to be taken in the design of models of care, such as integrated in existing services or health centres, semi-vertical clinics like the Tumainis, one-stop care centres or a more community-based model and should be done in collaboration with the target population as there is no one size fits all.

Distance and transportation costs were identified as barriers for survivors seeking care, and in addition to bringing the care closer to survivors, systems for ensuring transportation should also be considered. However, this is not simple, as ensuring transportation for survivors can compromise confidentiality and further expose and harm survivors. Cash reimbursement might be an alternative but has to be implemented with great care to mitigate risk of misuse and exploitation, ensuring that safeguarding is at the heart of the activity.

### Methodological considerations

This study has strengths as well as limitations. The three authors leading the FGDs have extensive experience of working with survivors of SV and IPV within the study area. Both EKK and NHG are Congolese nationals with a deep understanding of the cultural context in North Kivu, and HR had been working and living in North Kivu for more than 12 months prior to the data collection and had conducted several interviews, group discussions and community assessments in the area. However, the fact of being an “outsider” can never be completely disregarded and this might have impacted the discussions. Among the other research team members (note takers and translators) the majority were Congolese and one were of another nationality.

Both convenience and purposive sampling methods were used, and gatekeepers in the specific villages were used to identify participants. This might have introduced a bias in the selection of participants. By working in catchments areas of MSF programmes there is a risk that participants may have more awareness and knowledge of this topic. In addition, the use of gatekeepers could introduce power imbalances leading to social desirability bias as well as potentially limiting the diversity of participation. To mitigate these factors a diversity of gatekeepers were used. The aims of the project were thoroughly discussed, and the voluntary nature of participation was clearly communicated both to gatekeepers and participants.

The fact that all members of the research team were working for the humanitarian organisation MSF, active in the study area, might have increased the risk of social desirability bias and collaborative consensus bias. To minimise the risk of desirability bias the research team avoided questions about how the individual participants would act, but focussed rather on how other community members would behave in case of sexual violence and a vignette was used for this purpose. The fact that the research team was working for MSF can have had an impact on the discussions, especially those concerning the services available. However, our impression is that participants could speak freely and we received both negative and positive feedback, but this fact needs to be taken into consideration when interpreting the results. Moreover, there is inevitably a power imbalance between the participants and the facilitators. To address this, care was taken to explain the voluntary basis of participation and that there was no impact on the aid provision for individuals or the community based on the participation or the views expressed during the discussions.

Several data collectors were involved, as well as note takers and translators, and discussions were led by three different members of the research team. This might have had an impact on the nature of the discussions. However, the use of a topic guide and the same vignette in all discussions helped ensure a consistent approach across discussion groups and that the different topics of interest were addressed. The vignette was a story of a female survivor and the participants were later asked to reflect around what would be different if the survivor was a man or a child. This might have focussed the discussions more on female survivors.

The FGDs were conducted separately by gender to allow participants to be able to speak more freely about this sensitive topic, but the research team (FGD leader, note takers and translators) did include both males and females for most of the discussions and the FGD leaders were not always of the same sex as the participants in the group which might have had a negative impact. For the same reason we also aimed to create focus groups based on age similarity among the participants, but this was not possible for all FGDs, which might also have had an impact on the discussions.

In addition to the FGD leaders there were additional research team members present, one for translation and one for note taking and additional support/input. Having several members of the research team present might impact the sense of speaking feely and efforts were made to ensure a trusting and relaxed atmosphere where the participants could feel comfortable speaking.

To enhance confirmability, the facilitators maintained an ongoing dialogue throughout the data collection process [42]. All facilitators participated in the data analysis, to ensure that the interpretations reflected a collective understanding among both the data collectors and colleagues who were not directly involved in the data collection. Additionally, the involvement of multiple team members, continuous dialogue and explicit description of the research team’s roles and positions contributed to the study’s reflexivity [42].

Effort was made to enhance transferability by providing a detailed description of the study’s context and of the analysis process followed [43]. However, as the data were collected from only two areas in different parts of North Kivu, the findings may not be fully transferable to other settings.

In-depth analysis of gender, age, socioeconomic status, disability, diverse sexual orientation or gender identities or the intersection between these factors, was not possible due to lack of information about these aspects. Thus, the methodology did not allow for further exploration of potentially marginalized groups. The lack of an intersectional analysis may pose some limitations. Despite these limitations, we believe that this study provides valuable insights concerning community perceptions around access to care for survivors of sexual violence in the study area.

## Conclusion

Even though the community members recognise the importance of seeking care after the experience of sexual violence, it is evident that survivors face a plethora of different barriers and that many survivors will never seek care. Misconceptions and a high degree of focus on the physical consequences of sexual violence can limit care seeking, and more awareness raising, focusing on the broader consequences and the opportunities to receive both medical and psychosocial support, is needed. While community and family attitudes and norms may prevent survivors from seeking care, there is also a great potential for the community to provide support for survivors, an aspect that needs to be addressed in programmes targeting sexual violence in the area. Services should be adapted to different groups of survivors, to ensure that healthcare workers are supportive and that services are provided with discretion. Addressing access to care is crucial when setting up care for SV survivors and must be a core part of the activities. Awareness raising, community engagement, transforming harmful attitudes and norms, addressing stigma, training of healthcare workers and careful design of models of care in collaboration with the target population, are key strategies for improving access. Further research is needed to explore barriers and enablers for specific vulnerable groups as well as the intersection between different demographic and individual factors. Future research efforts should also aim at exploring the perspectives of survivors of sexual violence, including minors, and the perspectives of healthcare providers in the area.

## Data Availability

No datasets were generated or analysed during the current study.

## Abbreviations

DRC: the Democratic Republic of the Congo
FGD: Focus group discussion
IPV: Intimate partner violence
MSF: Médecins Sans Frontières
STI: Sexually transmitted infection
SV: Sexual violence
